# Study protocol for feasibility and safety of adopting early oral feeding in post total laparoscopic total gastrectomy (overlap esophagojejunostomy): A multicentre randomized controlled trial

**DOI:** 10.3389/fnut.2022.993896

**Published:** 2022-08-23

**Authors:** Jun Yang, Qinchuan Yang, Weidong Wang, Xiaoyan Chai, Haikun Zhou, Chao Yue, Ruiqi Gao, Zhenchang Mo, Panpan Ji, Danhong Dong, Jiangpeng Wei, Jinqiang Liu, Ying Zhang, Xiaohua Li, Gang Ji

**Affiliations:** ^1^Department of Gastrointestinal Surgery, Xijing Hospital, Air Force Military Medical University, Xi’an, China; ^2^Department of Radiotherapy, Xijing Hospital, Air Force Military Medical University, Xi’an, China

**Keywords:** total laparoscopic total gastrectomy, overlap esophagojejunostomy, early oral feeding, delay oral feeding, oral nutritional supplements

## Abstract

**Background:**

Total laparoscopic total gastrectomy (TLTG) for gastric cancer, especially with overlap esophagojejunostomy, has been verified that it has advantages of minimally invasion, less intraoperative bleeding, and faster recovery. Meanwhile, early oral feeding (EOF) after the operation has been demonstrated to significantly promote early rehabilitation in patients, particularly with distal gastrectomy. However, due to the limited application of TLTG, there is few related research proving whether it is credible or safe to adopt EOF after TLTG (overlap esophagojejunostomy). So, it is urgent to start a prospective, multicenter, randomized clinical trials to supply high level evidence.

**Methods/design:**

This study is a prospective, multicenter, randomized controlled trial with 200 patients (100 in each group). These eligible participants are randomly allocated into two different groups, including EOF group and delay oral feeding (DOF) group after TLTG (overlap esophagojejunostomy). Anastomotic leakage will be carefully observed and recorded as the primary endpoints; the period of the first defecation and exhaust, postoperative length of stay and hospitalization expenses will be recorded as secondary endpoints to ascertain the feasibility and safety of adopting EOF after TLTG (overlap esophagojejunostomy).

**Discussion:**

Recently, the adoption of TLTG was limited due to its difficult anastomotic procedure, especially *in vivo* esophagojejunostomy. With the innovation and improvement of operating techniques, overlap esophagojejunostomy with linear staplers simplified the anastomotic steps and reduced operational difficulties after TLTG. Meanwhile, EOF had received increasing attention from surgical clinicians as a nutrition part of enhanced recovery after surgery (ERAS), which had shown better results in patients after distal gastrectomy. Considering the above factors, we implemented EOF protocol to evaluate the feasibility and safety of adopting EOF after TLTG (overlap esophagojejunostomy), which provided additional evidence for the development of clinical nutrition guidelines.

**Clinical trial registration:**

[www.chictr.org.cn], identifier [ChiECRCT20200440 and ChiCTR2000040692].

## Background

According to Global Cancer Statistics 2018, gastric cancer remained the fifth most common cancer, especially in East Asia, Eastern Europe, South Africa and certain Western Asian countries (1). Laparoscopic distal gastrectomy, as one of the radical surgical procedures, has gained wide approval with technical feasibility and oncological safety (2). However, for cT_2_ or deeper tumors, total gastrectomy should be considered, especially the upper gastric cancer, middle or lower gastric cancer (proximal margin exceeding 3 cm with an expansive growth pattern or 5 cm for those with an infiltrative growth pattern) (3).

Nevertheless, the promotion of total laparoscopic total gastrectomy (TLTG) has been limited due to the technical difficulty of esophagojejunostomy. In recent years, various types of *in vivo* esophagojejunostomy techniques have been innovated and improved, which reduce the difficulty of operation and simplify the operation steps. In particular, the overlap esophagojejunostomy with linear staplers in total gastrectomy changed the direction of the jejunal limb and alleviated tension at the anastomosis, which effectively prevented postoperative complications and achieved satisfactory postoperative results (4).

Inevitably, total gastrectomy results in the loss of the nutrient absorption function of the stomach, leading to postoperative malnutrition. Therefore, more and more studies have focused on perioperative nutritional therapy for gastric cancer. The 2006 ESPEN Guidelines on Enteral Nutrition suggested initiating normal diet or enteral intake early after (Grade A) (5). Additionally, early oral feeding (EOF), as part of enhanced recovery after surgery (ERAS), has shown beneficial clinical results after open gastrectomy, especially for patients after distal gastrectomy (6–11). Several studies have reported that EOF after distal gastrectomy reduces postoperative hospital stay, medical costs, and complications (2, 12). Regrettably, regarding total gastrectomy, only few studies assessed the safety and feasibility of EOF in post TLTG (overlap esophagojejunostomy) due to the limited application of TLTG (3).

On the other hand, oral nutritional supplements (ONS), as a form of enteral nutrition, have attracted attention for its ability to reduce the incidence of postoperative complications. The results of a randomized clinical trials conducted by Meng Q showed that: compared with patients who received a conditional postoperative diet, patients receiving ONS had dramatically less fatigue and appetite loss (13). However, there is a great heterogeneity in clinical studies regarding the choice and effectiveness of ONS. Meanwhile, patient adherence rates to ONS were unsatisfactory at a low 42% (14, 15).

Based on these deficiencies, we hypothesized that patients with EOF would fall in fewer complications and get recovery earlier than delay oral feeding (DOF) after TLTG (overlap esophagojejunostomy). In this study, we designed a protocol to evaluate the feasibility and safety of adopting EOF after TLTG (overlap esophagojejunostomy) and provided more clinical evidence for clinical nutrition guidelines.

## Methods and designs

Patients were evaluated according to relevant clinical parameters (serological tumor markers, CT and/or MRI, ultrasound endoscopy, nutritional indicators, etc.) prior to treatment. We planned to recruit 200 patients who are eligible for TLTG (overlap esophagojejunostomy). After signing an informed consent form, they were randomly assigned to the trial group (EOF) or the control group (DOF) in a 1:1 ratio. The randomization sequence was generated by a statistics professor using SPSS 28 software. The randomization sequence numbers were kept in opaque envelopes by a dedicated person.

To avoid selection bias, personnel associated with the randomization process will not be directly involved in this study.

## Aim/purpose

This trial is a multicentre, prospective clinical study intended to implement an EOF protocol in patients after TLTG (overlap esophagojejunostomy). The trial has been registered at http://www.chictr.org.cn (ChiCTR2000040692).

## Study subjects/population

The subjects of this trial are patients with gastric cancer who will receive TLTG with overlap esophagojejunostomy. After signing an informed consent form, they received a random serial number. All processes strictly followed the provisions of the Ethical Review of Biomedical Research Involving Humans (Trial), the Declaration of Helsinki v.08, and the International Ethical Guidelines for Biomedical Research Involving Humans.

## Patients selection

### Inclusion criteria

1. Patients who aged between 18 and 65;
2. Patients who are diagnosed with gastric cancer through upper gastrointestinal endoscopy;
3. Patients who meet the surgical requirements for TLTG;
4. Patients who have not undergone anti-tumor therapy, such as chemotherapy or radiotherapy, prior to the operation;
5. Patients with the Eastern Cooperative Oncology Group (ECOG) performance status ≤ 2;
6. Patients with American Society of Anesthesiologists (ASA) physical status < 3;
7. Patients with the nutrition risk screening 2002 (NRS2002) score ≤ 2;
8. Patients who sign the informed consent for research.

### Exclusion criteria

1. Patients diagnosed with multiple tumors;
2. Patients who have done stomach related surgery;
3. Patients with an emergency situation need to deal, such as gastrorrhagia, pyloric obstruction or gastrointestinal perforation;
4. Patients who are intolerant of enteral nutrition in postoperative of days (POD);
5. Patients with psychiatric disorders;
6. Pregnant or lactating women;
7. Patients who do not agree with research treatments or TLTG operation.

### Sample size

We designed a non-inferiority clinical trial, aiming to verify that EOF protocol in patients after TLTG (overlap esophagojejunostomy) is not inferior in terms of anastomotic leak rate. According to data from previous studies (16), the incidence of anastomotic leak in gastric cancer was 1.9%. Therefore, considering a non-inferiority margin of 5% (α = 0.05, β = 0.20, δ = 0.05, 80% power, 10% dropout rate) and secondary study indicators, such as intestinal intolerance, we decided to recruit 200 patients with at least 100 cases in per group.

### Participating entities

This was a multicentre, prospective, randomized controlled trial with six participating medical institutions (Xi-Jing Hospital, Tang-Du Hospital, First Affiliated Hospital Xi’an Jiaotong University, General Hospital of Ningxia Medical University, Henan Provincial People’s Hospital, The First Affiliated Hospital of Shanxi Medical University). The method of recruitment was competitive. All research institutions and personnel were approved by the Ethics Committee. The doctors and nursing team had rich experience in gastric cancer and postoperative nutritional treatment.

### Study approach/randomization

**FIGURE 1:**
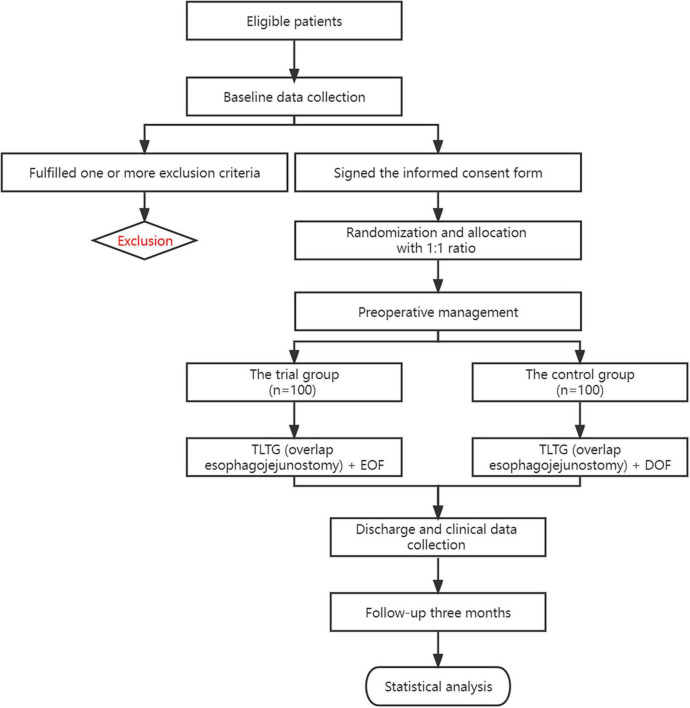
Study design flow chart.

Each trial/control group was randomly divided into 100 eligible patients. After applying the screening criteria, stratified random grouping will create an experimental group, who will adopt TLTG (overlap esophagojejunostomy) + EOF; a control group, who will undergo TLTG (overlap esophagojejunostomy) + DOF. The research flow chart is summarized in Figure 1.

### Ethics and supervision

The trial procedures and informed consent form were approved by the Chinese Clinical Trial Registry and the registration number was ChiECRCT20200440. Any adverse events (AEs) occurring were immediately submitted to the research leaders and the Committee; Any severe adverse reactions were reported to the Committee to determine whether the test should be stopped.

### Surgical procedure

**FIGURE 2:**
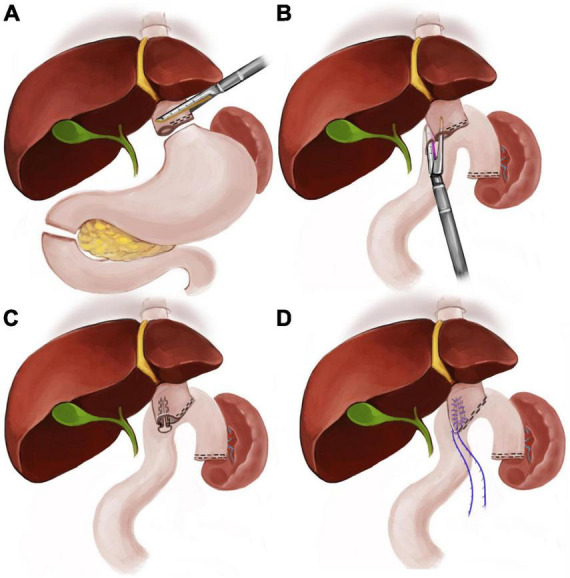
The surgical diagram of the esophageal-jejunal anastomosis **(A)**. The posterior wall of the stump of the esophagus will be fenestrated to facilitate the anvil insertion of the linear stapler for further anastomosis **(B)**. The esophagus will be anastomosed with the severed jejunum with using a 45 mm linear stapler **(C,D)**. The inner layer of the common opening of the esophagus and jejunum will be closed and sutured with absorbable barbed wire (V-LOC).

The surgical procedures will be performed by experienced surgeons. According to clinical guidelines for the diagnosis and treatment of gastric cancer, Roux-en-Y reconstruction was established by esophageal jejunostomy (overlapping anastomosis) after completion of total gastrectomy with D2 lymphadenectomy (17). The front of the esophageal hiatus of the diaphragm and the foot of the left diaphragm will be opened, and the lower part of the esophagus will be fully freed. The esophagus will be transected with a 60 mm-laparoscopic linear stapler and open a small hole on its right side. The jejunum will be transected 25 cm from the ligament of Treitz, and a small hole will be made at the mesangial margin of the distal jejunum 6 cm from the transected end by an ultrasonic scalpel. The distal jejunum will be lifted, and one fork of the 60 mm-laparoscopic linear stapler will be carefully inserted into the hole of the esophagus under the guidance of nasogastric tube, as the other fork will be inserted into the oral side of the jejunum. The common opening will be closed manually under a microscope, and jejunal side-to-side anastomosis will be performed 50 cm distal to the esophageal-jejunal anastomosis. The surgical diagram of overlap anastomosis performance is showed in Figure 2.

### Nutritional therapy

According to the Harris-Benedict formula in the ERAS protocol, the daily energy (20–25 kcal/kg⋅d) and protein (1.0–1.2 g/kg⋅d) consumption for 1 week after surgery is calculated for each patient. Based on reported studies and consumption requirements, we established a dietary schedule (18, 19). Each phase of the diet and the patient’s response is closely monitored by a professional dietitian and care team. If necessary, for patients with poor nutritional status in the two groups, parenteral nutrition tubes should be placed to ensure adequate calorie intake after surgery. The intervention measures and observation indicators are intimately shown in Table 1.

**TABLE 1:** The intervention measures and observation indicators.

	Period of study							
	Enrolment	Allocation	Postoperation					Endpoint
TimePoint	Day-7∼0	OP day	POD1	POD2	POD3	POD4	POD5-7	
**Enrollment**								
Eligible patients	×	×						
Inform consent	×	×						
Endoscopy and body examination	×							
Baseline	×							
Allocation	×	×						
**Intervention**								
EOF		Sips of water 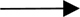	Meglumine diatrizoate esophagogram, liquid diet, ONS	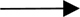 soft diet and ONS		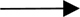		normal diet
DOF			Intravenous fluids, until the appearance of intestinal exhaust or bowel sounds		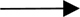	sips of water, liquid diet and ONS	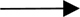 soft diet 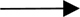	normal diet
**Assessment**								
Time to recovery of gastrointestinal function			×	×	×	×	×	
Nutritional markers	×		×		×		×	
Gastrointestinal hormone levels	×		×		×		×	
Diet intake			×	×	×	×	×	×
Tolerance of oral feeding			×	×	×	×	×	
Postoperation complication			×	×	×	×	×	×
Extent of weight change	×						×	×
Postoperative LOS							×	×
Hospital expense							×	×

### Early oral feeding group

The EOF group will receive EOF protocol: (1) Day of surgery: a few sips of water (< 50 mL/h) 6 h after surgery; (2) POD 1: performing a meglumine diatrizoate esophagogram to rule out anastomotic leak; encouraging oral administration of 500 mL liquid diet in 5 divided doses, including 10% glucose saline and ONS in absence of intestinal intolerance; and (3) POD2-7: starting soft diet and ONS.

During postoperative dietary management, soft diet is given gradually according to the patient’s physical status until at least 60% of normal food intake is restored.

### Delay oral feeding group

The participants randomized to the DOF group (control group) will receive the same oral feeding content as described above during this trial (starting on POD4). The only difference between the trail and control groups will be the oral feeding start time after surgery: (1) POD1-3: the DOF group will receive 40–50 ml/kg⋅d of intravenous fluids postoperatively to fulfill the basic consumption and fast for 3?days; (2) POD4-7: they receive a few sips of water on POD4. Then they resumed liquid diet, ONS and soft diet; No additional nutritional supplementation (such as enteral tube feeding) would be given.

### Nutrition therapy notes

1. Controlling the temperature of ONS at 37–39^°^C to avoid digestive discomfort;
2. Reminding patients to maintain proper sitting posture, avoiding accidental aspiration;
3. Varying the consistency of ONS according to the patient’s eating habits;
4. Giving smaller amounts at a time to prevent swallowing fatigue;
5. Avoiding food residue in the mouth and maintaining clean oral hygiene;
6. Making dynamic adjustments to oral intake. Daily caloric and protein requirements were met when approximately 60% of the meal can be consumed normally.

All groups were given ONS (Prosure, powder, Abbott, Spain, 380 g/can, EPA 1.06 g per serving size) at 240 ml per day during the period oral feeding, in addition to standard diet (or instead of being part of a normal diet). We regulated the time and limited intake of ONS by measuring cup. The daily intake will be divided into 5–6 portions. The nutritional information of the Prosure supplement in this trial is shown in Table 2.

**TABLE 2:** The nutritional information of the Prosure® supplement.

Nutrient composition of prosure®		
Nutritional ingredient	Amount per 240 ml (302 kcal)	Unit
Protein	15.97	g
Fat	6.14	g
Carbohydrate	43.24	g
Fiber	4.97	g
Fructo-oligosaccharide	2.64	g
Eicosapentaenoic acid	1.06	g
Docosahexaneoic acid	0.48	mg
**Vitamins**		
Vitamin A	324	μgRE
β-carotene	168	μgRE
Vitamin D3	4.07	μg
Vitamin E	48.3	mgα-TE
Vitamin C	103.2	mg
Vitamin B1	0.6	mg
Vitamin B2	0.7	mg
Vitamin B6	0.72	mg
Vitamin B12	1.32	μg
Folic acid	76	μg
Niacin	2.88	mg
Pantothenic acid	2.3	mg
Biotin	12	μg
**Minerals**		
Na	288	mg
K	432	mg
Cl	336	mg
Ca	348	mg
P	160	mg
Mg	100.8	mg
Zn	6	mg

### Tolerance criteria

According to the 2016 SCCM/AS-PEN nutrition guidelines, these include the following (18).

1. Abdominal distension or abdominal pain;
2. Nausea or vomiting;
3. Diarrhea.

If any of the above symptoms occur, it was considered intolerance of the intestine. For patients with mild symptoms, symptomatic management was indicated; for patients with severe anastomotic leak or those who cannot meet 60% of their nutritional and energy requirements, parenteral nutrition was recommended.

### Discharge criteria

1. Returning to normal bowel habits;
2. Maintaining normal body temperature for 3 days without postoperative complications;
3. No need for intravenous drugs or nutritional fluids;
4. Independent activities;
5. Eating and tolerating more than 60% normal diet.

### Peri-operative procedures

Before surgery, eligible patients will undergo gastroscopy and CT scans to evaluate the location and size of gastric carcinoma, but patients with metastatic tumors will be excluded according to the assessment of two experienced pathologists. All patient clinical pathways will follow Surgical Residents’ Postoperative Practices and Barriers and Enablers to the Implementation of an ERAS Guideline, which recommends that patients accept preoperative education and pre-emptive and multimodal analgesia. The same senior gastrointestinal surgery team is responsible for TLTG. After stomach resection, total laparoscopic oesophagojejunostomy will be performed following the total gastrectomy. The EOF plan is personalized and adjusted dynamically every day by the nutritionist to ensure adequate intake of calories and protein.

### Study endpoints

#### Primary study endpoints

The primary outcome measure is the incidence of anastomotic leakage after the surgery.

#### Secondary study endpoints

The secondary outcome measures include postoperative complications, such as infection, internal hemorrhage, postoperative paralytic ileus, pulmonary complications, and other organ complications occurred in 1 month. Secondary indicators will also include the time of first exhaust and defecation (days), tolerance of oral feeding, nutritional markers, gastrointestinal hormone levels, duration of postoperative hospital stay (days) and hospitalization expenses (yuan).

#### Data collection and schedule

According to the informed consent form, the researcher collected various clinical information from the patient. Data collection included general information, previous surgical history, NRS 2002 score, ASA score, BMI, prevalence and classification of all postoperative complications, tolerance of oral feeding (according to 2016 SCCM/AS-PEN Nutrition Guidelines), nutritional markers (transthyretin, serum lymphocyte count, serum albumin and pre-albumin levels), gastrointestinal hormone levels (gastrin and kinesin levels), time to recovery of gastrointestinal function (first exhaust and defecation), extent of weight change, duration of postoperative hospital stay and cost-effectiveness indicators (hospital costs).

The experimental data shall be recorded in the CRF table by the treatment doctor, and the data records shall be complete, timely, accurate and true. Patient data recording and modification will be performed in the research center, and the CRF tables of each subject will be reviewed and signed by the researchers at the sub-center. The monitor will review and observe each item of data in the CRF table. Patients are followed up for 1–3 months *via* WeChat or phone after discharge.

#### Statistical analysis

To explain the differences in the safety of EOF and DOF for patients after TLTG (overlap esophagojejunostomy), the research team will compare the incidence of anastomotic leakage and postoperative complications, gastrointestinal motility, and hospitalization by using a non-inferiority test between the two groups.

All obtained data will be statistically analyzed using SPSS 28.0 statistical software and GraphPad Prism 8. Continuous data will be expressed as the mean ± standard deviation (−X ± S); measurement data will be assessed by *t*-test; and enumeration data will be assessed by the X^2^ test. *P* < 0.05 will be used as the significance level. Considering the complications after the treatment and patient adherence rates to ONS at a low 42%, we used intentional analysis for this study to maintain the effect of randomization.

## Discussion

Epidemiologic studies have shown that the incidence of upper and middle digestive carcinoma requiring total gastrectomy has increased in recent years (20). However, the adoption of TLTG is limited due to its difficult anastomotic technique, especially *in vivo* esophagojejunostomy. Overlap, as a modified esophagogastric anastomotic procedure, maintains normal peristaltic direction and reduces tension on the esophageal-jejunal anastomosis, especially in the laparoscopic total gastrectomy with linear staplers (3, 4).

Total gastrectomy, despite being a radical treatment for gastric cancer, inevitably results in the loss of nutritional function of the entire stomach (21–26). Therefore, increasing researches had focused on the perioperative nutritional treatment of gastric cancer. EOF, as part of ERAS nutrition, has shown better results in patients after distal gastrectomy (9–12), including a reduction in postoperative hospital stay, medical costs and complications. Compared with DOF, many researches have shown significant benefits of EOF: stimulating intestinal motility, accelerating the recovery of postoperative bowel function, providing overall protein to the intestinal mucosa, reducing the incidence of intestinal flora imbalance and thus decreasing the rate of complications (6, 9, 25, 26). Regarding total gastrectomy, however, due to the limited application of TLTG, there were few studies on EOF after TLTG (overlapping esophagojejunostomy).

ONS, as a form of enteral nutrition, has attracted attention for its ability to reduce the incidence of postoperative complications (25). Nevertheless, regarding the selection of the type of ONS, there is a large heterogeneity in the results of clinical studies, and a uniform standard has not yet been established. In addition, the price of nutritional preparations is generally high, and coupled with the current medical reimbursement policy, patients are not fully reimbursed for ONS after hospital discharge. Thus, patient compliance with ONS is not satisfactory (20).

Considering these objective factors, we attempted to implement an EOF protocol in patients undergoing TLTG (overlap esophagojejunostomy). Based on this clinical nutrition protocol, we established a multicentre, prospective, randomized controlled trial: To evaluate the feasibility and safety of adopting EOF after TLTG (overlap esophagojejunostomy). We will continue to explore and conclude the postoperative nutrition protocol for patients with gastric cancer. If the desired clinical outcomes are achieved, this will provide additional evidence-based medical research for the development of clinical nutrition guidelines.

## Ethics statement

The studies involving human participants were reviewed and approved by the Chinese Ethics Committee of Registering Clinical Trials. The patients/participants provided their written informed consent to participate in this study. Written informed consent was obtained from the individual(s) for the publication of any potentially identifiable images or data included in this article.

## Author contributions

YZ, XL, and GJ designed the research orientation and approach and approved the final manuscript. JY and QY wrote and submitted the manuscript. JY and WW completed the research ethics registration. GJ checked the measurement time and details of the trial. JW, DD, JL, PJ, RG, CY, ZM, HZ, and XC were responsible for the observation of patients in hospital. All authors have read this manuscript carefully and assented and consented the contents of the manuscript.
